# Processing False Beliefs in Preschool Children and Adults: Developing a Set of Custom Tasks to Test the Theory of Mind in Neuroimaging and Behavioral Research

**DOI:** 10.3389/fnhum.2020.00119

**Published:** 2020-04-15

**Authors:** Joanna Wysocka, Karolina Golec, Maciej Haman, Tomasz Wolak, Bartosz Kochański, Agnieszka Pluta

**Affiliations:** ^1^Faculty of Psychology, University of Warsaw, Warsaw, Poland; ^2^Bioimaging Research Center, World Hearing Center, Institute of Physiology and Pathology of Hearing, Warsaw, Poland

**Keywords:** theory of mind, false-belief task, neuroimaging, neurodevelopmental study, functional MRI (fMRI), functional near-infrared spectroscopy (fNIRS)

## Abstract

The theory of mind (ToM) is the ability to attribute mental states to others and is extremely important for social functioning. It has been widely examined in both behavioral and neuroimaging research, usually with the use of the many versions of the false-belief (FB) task. However, there is still not enough evidence from studies on the neurodevelopmental mechanisms of ToM mostly because of methodological limitations: lack of selectivity, mismatch of experimental and control tasks, and focusing on participants older than 6 years old. In the current study, we attempted to develop a computerized tool for ToM assessment suitable for both behavioral and neuriomaging testing in preschoolers. We designed a version of the classic change-of-location task with custom visuals and three fine-tuned conditions: FB, true-belief, and no-belief (NB). The usability of the task for further application in neurodevelopmental research was tested with three methods: first, behaviorally, with the use of a touch screen on a group of 75 children, followed by a functional MRI (fMRI) study on 13 adults, and a functional near-infrared spectroscopy (fNIRS) study on 19 preschool children. In line with our expectations, on the behavioral level, our task elicited the all-or-none performance in preschoolers. There was also a progression of performance with age in the FB condition. On the neural level, we observed the activation of structures involved in the ToM brain network in response to our task in both adults and children. The results therefore suggest that our task can be a useful tool for studying ToM development and its neural underpinnings.

## Introduction

The theory of mind (ToM) is the ability to infer mental states such as beliefs, intentions, or desires; it is crucial for understanding the behavior of others and adapting to social situations. ToM is typically measured with the false-belief (FB) task (FBT). In the classic FB paradigm, the participant is familiarized with a scenario in which the protagonist has incomplete knowledge about the situation she/he is involved in. Consequently, she/he has FBs concerning this situation. The participant is asked to efficiently predict the actions of the protagonist (Wimmer and Perner, 1983). Passing the FBT requires understanding that the mental states guiding the protagonist’s behavior may be inconsistent with reality or conflicting with the true beliefs (TBs) held by the participant as an independent observer. In the classic Sally–Anne unexpected transfer task, Sally places her toy in one location and the toy is moved by Anne during Sally’s absence. To complete the task, the participant must give an explicit verbal answer to the question of how the protagonist will behave, i.e., “Where would Sally look for the toy?” (Baron-Cohen et al., 1985). The verbal version of this task is believed to engage purposeful reasoning about others’ mental states, referred to as *explicit* ToM.

Performance on the verbal FBT is generally expected to be in accordance with the all-or-none model: the participant is either capable of FB reasoning and responds correctly or repeatedly indicates the wrong location (Baker et al., 2016). However, Bayesian change-point analysis of FBT behavioral performance by Baker et al. (2016) revealed that a short transition period within verbal ToM development may occur. It manifests as a “stably unstable” pattern of giving random responses.

Generally, the development of this ability measured with FBT has been shown to maintain a consistent path, as children’s answers become accurate between the ages of 3 and 5 years (Wellman, 2018). It is therefore believed that by the age of 5, children should reach a milestone and become able to take the perspectives of others: not only explaining others’ behavior as resulting from mental states but also recognizing that these mental states can be based on inaccurate or outdated information. Passing the FBT is thus interpreted as a standard indicator of such developmental achievement.

However, this inference has often been called into question. Classic FB paradigms have been criticized for their excessive complexity and executive function demands (Bloom and German, 2000; Rubio-Fernández and Geurts, 2013; see also Baillargeon et al., 2010; Devine and Hughes, 2014; Wellman and Cross, 2001, for meta-analytical reviews). First, reasoning about a linguistically elaborate storyline may be fairly challenging for younger children. Trying to follow the plot, they may become distracted from perspective taking. Second, more than one character is often present in a typical FB scenario. Although children are expected to track the mental states of the main character, an additional figure often participates by changing the location of the main character’s toy in the FB induction phase. Thus, in order to succeed, children need to switch between the perspectives of the characters and, additionally, inhibit their own perspective as observers. Last but not least, the role of the experimenter includes simultaneously presenting consecutive events, roleplaying the story, and, finally, introducing the test question, which may cause additional confusion and distract younger children from taking the main protagonist’s perspective. Finally, focusing on the object draws the child’s attention to its current (true) location (Rubio-Fernández and Geurts, 2013). Rubio-Fernández and Geurts (2013) demonstrated that if modified versions of the FBT are implemented with minimized linguistic, attentional, and executive demands, it becomes possible for 3-year-old children to succeed. Furthermore, using indirect, gaze-based measures within violation-of-expectation and anticipatory-looking paradigms, it has been demonstrated that it is already about or before the age of 12 months that children consider others’ beliefs when interpreting their behavior (for a review see Baillargeon et al., 2010). Present from the first year of life, the tendency to automatically take others’ beliefs into account, often referred to as *implicit* ToM, is nowadays widely studied using nonverbal unexpected change-of-location FBT (Kovács et al., 2010; Schneider et al., 2014). It is, however, far from clear to what extent implicit and explicit tasks engage the same mental mechanisms of ToM reasoning and constitute a continuous developmental path from the implicit tracking of others’ beliefs in the early stages of development to passing explicit, verbal versions of the FBT (Apperly and Butterfill, 2009; Heyes, 2014; Helming et al., 2016; Wang and Leslie, 2016; Southgate, 2018; Wellman, 2018; Haman, 2019).

Further research with the use of neuroimaging techniques is likely to provide substantial insight into the explanatory paths of ToM development. The results of studies to date mostly appear to demonstrate that the neural correlates of both verbal and nonverbal ToM task performance comprise a complex ToM network constituting a part of the so-called human “social brain”: the temporoparietal junction (TPJ), precuneus, posterior superior temporal sulcus (pSTS), dorsomedial prefrontal cortex (dmPFC), and temporal poles (e.g., Dodell-Feder et al., 2011; Kovács et al., 2014; Bardi et al., 2016). Nonetheless, most of these studies of brain activity in ToM contexts were done with adults or children of at least 6 years old, who have already mastered FBT performance. There are few neuroimaging studies focusing on the transitional ToM development stage with the participation of children younger than 6 years old (for a review see Haman, 2019).

To our knowledge, there are only two functional MRI (fMRI) studies in which the ToM task performance of young children was assessed online, with parallel brain activity measurements. In an experiment by Gweon et al. (2012), children aged 5–11 listened to short stories during an fMRI scan. Each story focused on the protagonist’s thoughts, beliefs, and desires; their social environment; or the physical state of objects and events. Richardson et al. (2018) examined the ToM neural network with fMRI in a substantial sample of children aged 3.5–12 years old while they watched an animated movie. At the center of the plot were two categories of unique events concerning the protagonists’ internal states: (1) physical sensations, mostly pain; and (2) mental states, comprising not only beliefs but also desires and emotions. The level of FB understanding was additionally controlled outside the scanner (using illustrated storybooks and questions embedded in the plot) and afterward correlated with neural results.

In both of the experiments described above, the results point to ToM network specialization as a basic neurocognitive developmental mechanism of mentalizing. However, these procedures differed significantly from the classic visual change-of-location tasks, which are most often used in behavioral examination of ToM maturity (Wellman and Cross, 2001). It is also worth noting that various kinds of attribution were examined in the ToM condition alongside belief attribution, whereas it has been reported that neural correlates of belief attribution and emotion attribution do not entirely overlap (e.g., Zaitchik et al., 2010; Lee et al., 2016). Indeed, reliable discrepancies are observed in the response of ToM network regions to such localizer tasks, as they vary in matters of modality (written stories vs. animations), participation demanded (responding vs. passive watching), and cognitive processes engaged (belief attribution vs. mental state attribution; Jacoby et al., 2016).

There is also a group of studies in which the data concerning functional brain organization or anatomical discrepancies were correlated with behavioral ToM results. For example, Grosse Wiesmann et al. (2017) have observed increased connectivity and white matter volume in children up to 4 years old who pass the behavioral version of the FBT. Xiao et al. (2019) assessed changes of functional connectivity related to developmental progression of ToM competence in children aged 4–8 years using resting-state MRI. They observed the relationship between functional connectivity within the ToM network and the results of behavioral parent-report ToM measures. Despite the indisputable importance of these findings for further research, the crucial information on participants’ ToM task performance in the aforementioned experiments is not fully precise as it was collected indirectly and offline, independently of the neuroimaging procedure.

As fMRI may be difficult to use in studies of particular populations (e.g., younger children) due to relatively high noise, staying still and lying supine in a tight enclosed space is particularly demanding for children, a growing amount of evidence in the area of developmental neuroscience is being collected with the functional near-infrared spectroscopy (fNIRS) method (Gervain et al., 2011). Both fNIRS and fMRI aim to measure changes in blood oxygen level-dependent (BOLD) signal and have been proven to deliver comparably reliable measures of hemodynamic responses in cortical brain regions (Balardin et al., 2017). Hyde et al. (2015, 2018) used fNIRS to record signals from ToM-related brain regions first in adults and later in 7-month-old infants using a similar procedure. Participants watched video stimuli which were previously used in behavioral experiments on implicit ToM (Southgate et al., 2007): a character holding an FB, portrayed by an actress, tries to retrieve a toy hidden in one box but which has been secretly transferred to another location by a puppet. The perspective of the character could therefore be disrupted by tracking the actions of the puppet. However, as well as the FB condition, Hyde et al. used an analogous TB control trial in which the transfer of the toy was observed by the protagonist. In contrast to FBs, TB understanding does not require noticing and resolving the conflict between the actor’s belief and reality. Including both the FB and TB conditions in the experimental paradigm enables us to identify brain regions specifically engaged in belief attribution processes and therefore meet the criteria of generality proposed by Saxe et al. (2004). According to these authors, defining a given brain region as functionally specialized in belief processing requires this region to respond to the stimuli provoking belief attribution in general (generality criterion). Moreover, the observed response has to be specific to belief attribution (specificity criterion). In brain regions fulfilling the generality condition, we would therefore expect a significant increase of activity for belief stimuli (for both TBs and FBs). Such an increase should be greater for FBs and should not occur for no-belief (NB) events (Sommer et al., 2007). In the case of the aforementioned experiment, increased activity associated with belief tracking was observed in the TPJ, showing no higher response for control conditions. However, there still remains the problem of specificity: we should not observe a greater response to non-mental stimuli within ToM network regions. To our knowledge, there have been no studies of neural correlates of FB reasoning in preschoolers in which both issues of generality and specificity have been appropriately addressed. This could be achieved by contrasting the FB condition both with a TB condition and a non-belief-related condition (NB) in which no mental state attribution is required to follow the plot.

In conclusion, in many cases, the reasoning about the neurodevelopmental mechanisms of ToM in the transitional stage seems to be of questionable validity. First, there are very few neuroimaging studies of children younger than 6 years old. It remains quite challenging to properly measure functional brain activation in younger participants, although doing so would undoubtedly shed light on the developmental mechanisms of ToM. Second, most of the results are derived from neuroimaging studies applying methodologically incomparable mentalizing and control tasks. The measures of brain structure and activity are often contrasted with the results of a behavioral ToM battery, which is usually applied independently of the neuroimaging procedure so that no data on the scope of neural activity concerning FB reasoning and control trials (TB, NB) are collected online. Finally, even if the FBT occurs simultaneously with the brain function measurement, it is rarely compared to both TB and NB control trials.

The overall objective of the current study was to develop a carefully controlled FBT with the following features: (1) child-friendly stimuli; (2) it is equally well-adjusted to computerized behavioral testing and neuroimaging techniques; (3) precisely matched experimental (FB) and control (TB, NB) conditions; (4) minimization of executive; and (5) linguistic demands; and (6) provides a measure resistant to repeated tests (which are indispensable in neuroimaging studies) which should be able to identify the all-or-none pattern of choices and the developmental progression found in previous research with explicit FBTs. Additionally, the task should be suitable for testing both implicit and explicit ToM reasoning in children (with only minimal adjustments). The custom designed version of the verbal ToM task was then tested with three methods: behaviorally in children of preschool age (Study 1), with fMRI in adults (Study 2), and finally with fNIRS in children aged 3–5 (Study 3).

While the comparison of explicit and implicit task equivalents became our future goal (see “Summary and Future Prospects” section), the current study focuses only on the explicit version of the task, which should lead to results similar to those of previously studied FBTs. We assumed, then, that the indicators that the goal had been achieved would be 3-fold. First, on the behavioral level: (1a) all-or-none performance of the preschoolers on our custom ToM tasks; (1b) a typical progression of performance between the age of 3 and 5 on the verbal FBTs, but with no significant age differences in performance of the control tasks. Second, (2) on the neural level, greater BOLD signal changes within cortical regions of the ToM network for the belief conditions, in contrast to NB events, with the highest response being in the right TPJ (rTPJ) for FB trials. Third, (3) a comparable pattern of neural responses between children and adults across the two imaging modalities (fMRI and fNIRS) in FB and NB conditions. We also expected to find weak-to-moderate correlation between performance in ToM tasks and linguistic (syntactic) competence and inhibitory control (inhibition of reaction) abilities.

## Study 1

### Materials and Methods

#### Participants

A total of 75 typically developing children (aged 3.3–5.11 years, *M* = 4.55; SD = 0.81; *n* = 22 3-year-olds, *n* = 27 4-year-olds, *n* = 26 5-year-olds; *n* = 42 boys, *n* = 33 girls) were tested in the behavioral study. They were recruited from three local kindergartens in Warsaw and nearby areas. Written informed consent was obtained from the parents/caregivers of the children participating in the study. All children assented to taking part in the experiment. Recruitment and experimental protocols were approved by the Ethics Committee of the University of Warsaw and conducted in accordance with the World Medical Association’s Declaration of Helsinki.

#### Theory of Mind Task

The task was designed based on the standard unexpected transfer procedure originating from Wimmer and Perner (1983), later adopted and largely simplified by Baron-Cohen et al. (1985) and Baillargeon et al. (2010). It was created using some modifications suggested by Rubio-Fernández et al. (2017).

**Figure 1:**
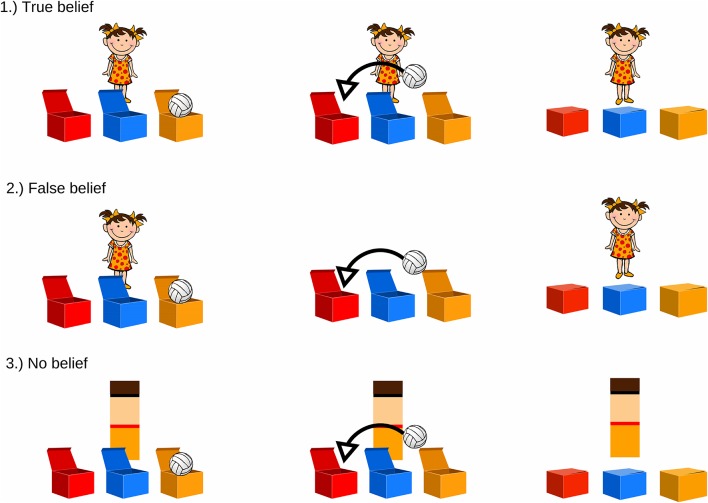
Examples of theory of mind (ToM) tasks. Each participant watched four cartoons of each condition. (1) True-belief (TB) and (2) false-belief (FB) conditions differed in the presence of the character while the toy changed location. In the (3) no-belief (NB) condition, the character was replaced with a color-matched geometrical shape.

The ToM task stimuli set contains short cartoon clips which consist of illustrations depicting a child (nine characters differing in hair and clothing colors: boys with short hair, in trousers and a t-shirt; girls wearing pigtails and a dress) and a toy (teddy-bear/ball/doll/car/truck/rubber duck). There are three boxes in front of the character, differing in color and position across trials. Each cartoon is assigned to one of two conditions depending on whether the character’s belief is consistent (TB) or inconsistent (FB) with the object’s actual location. Moreover, there is a control condition (NB), in which the character is replaced with a color-matched rectangle. All cartoons are balanced in terms of color scheme, total duration (35 s), and the presence of specific test events (change of toy’s location, movement of the character/rectangle, test question). They are presented in a random order four times within each condition (Figure 1).

In the FB condition, participants are presented with the character observing the toy being placed in the first box (the toy moves itself, without any interaction with any character). Once the toy is inside, the lids are closed, and the character turns around, moves to the left side of the screen, and leaves the scene (6 s). During his/her absence, the toy moves itself from one box to another, and the lids are closed again (13–16 s). Then, the character comes back (18 s), and the previously recorded test question is introduced (23 s): “*Where does the child think the toy is?*” In each condition, the scene is displayed for 10 s, and after that period of time, the next trial starts automatically.

In the TB condition, the scenario is identical to that described above, with one difference: the character leaves the scene and comes back immediately (6–10 s), so the change of the toy’s location is observed by the participant (18–22 s). The same test question is then asked (23 s): “*Where does the child think the toy is?”*

The NB condition, which is supposed to not induce any inferences about belief acquisition, does not contain a human character—they are replaced by a color-matched rectangle performing actions similar to those of the character (the rectangle disappears from the screen and appears again; 5–10 s). The change of the toy’s location occurs in the same manner as in the belief conditions (17–20 s). At the end of the cartoon, the test question is asked (23 s): “*In which box is the toy?*”

#### The Assessment of Language Skills

The Test of Language Development (Test Rozwoju Jezykowego, TRJ; Smoczynska et al., 2015) is the first normed Polish multi-scale test for pre- and early-school children aimed at the assessment of language skills. For the purposes of validation, only the “Grammar–sentence comprehension” subtest was used, as understanding complex sentences is one of the aspects of language believed to best explain the variation in FBT results (Milligan et al., 2007). Child participants were presented with 32 sets of four illustrations while an experimenter read sentences of varying grammatical complexity one by one. Each time they were asked to choose the picture which best fits what they heard, their accuracy was measured.

#### The Assessment of Executive Functions

The custom-designed Day–Night computer procedure (Garon et al., 2008) was used to assess inhibitory control in child participants. Subjects were asked to respond to the picture of day/night or summer/winter presented in the upper center of the touch screen. They were instructed to choose a picture of the opposite of the illustration presented, so they had to inhibit choosing a picture identical to the one presented at the top of the screen. Accuracy and reaction times were measured.

### Study 1 Procedure

This part of the testing took place in local kindergartens. Children sat in front of a laptop equipped with a touch screen. At the beginning, the experimenter gave the children some short training in order to familiarize them with the experimental situation and ensure that they understood the task. Participants were informed that they would soon watch funny cartoons and solve some riddles. First, they performed training trials presented in pseudorandom order (three FBs, three TBs, and three NBs) in which the experimenter asked leading questions (“Look, what is happening now?”; “Is the boy/girl here right now?”; “Does the boy/girl see what is happening? Why?”) and gave feedback so that the children could understand what is happening in the story and learn how to respond on the touch screen. After the last training trial, the experimenter announced that the child is now ready to do the task on their own, without any cues. The behavioral procedure consisted of four FB trials, four TB trials, and four NB trials presented in random order. Presentation^®^ software (Version 18.0, Neurobehavioral Systems, Inc., Berkeley, CA, www.neurobs.com) was used to display the stimuli on the screen. Reaction times and accuracy of the responses were collected. Afterward, children completed the Day–Night task and the Test of Language Development. Finally, they were rewarded with a colorful sticker and a t-shirt. The whole procedure lasted about 25–30 min.

### Study 1 Data Reduction and Analyses

Participants were classified into three groups depending on their accuracy in each condition of the ToM task. We assumed that even if children’s decisions are based on the all-or-none criteria, some deviant decisions—resulting from some uncontrolled factors and distractors—may still occur. We divided the probability space of the possible decision patterns into three categories with roughly equal probability assuming random decisions. Participants with three or four correct answers were classified as “Passers,” children who gave no more than one correct answer were counted as “Non-Passers,” while participants with two correct and two incorrect answers were classified as “Random” (randomly answering). The *Random* category may comprise children who did not understand the task, were distracted, or represent the short transitional stage according to the model by Baker et al. (2016). The chance level of probability of being classified to a given group was determined to be as follows: 31.25% for Passers and Non-Passers, 37.5% for Random. The analyses were conducted in the following steps:

1. Assuming all-or-none performance in the FBT, we expected the proportion of participants who choose at random to be significantly below chance distribution. This hypothesis was tested with binomial tests in all age groups separately.
2. To show developmental progression in the verbal FBT, chi-square tests with Yates’ continuity correction were computed on the proportion of Passers against Non-Passers in the three age groups. Children who were classified as Random were not included in this analysis.
3. Additionally, two one-way ANOVAs were conducted to compare results on the Test of Language Development and the Day–Night Task between age groups. Finally, Spearman’s Rho coefficients of correlation between accuracy levels in the ToM task, the Test of Language Development, and the Day–Night Task were computed.

SPSS and R Statistics were used for the behavioral data analyses (IBM Corp. Released 2016).

### Study 1 Results

The first analysis found that only a few children were classified as Random across the three age groups (Table 1). All tests against chance level (37.5%) were highly significant: 3-year-olds binomial two-tailed *p* = 0.0066, 4-year-olds *p* = 0.022, 5-year-olds *p* = 0.001.

**Table 1:** The proportion of Passers, Non-Passers and Random in each theory of mind (ToM) task condition, broken down by age.

	True Belief	False Belief	No Belief
	3 years old		
Passers	*n* = 17 77.3%	*n* = 2 9.1%	*n* = 20 90.9%
Random	*n* = 4 18.2%	*n* = 2 9.1%	*n* = 2 9.1%
Non-Passers	*n* = 1 4.5%	*n* = 18 81.8%	*n* = 0 0%
			Total = 22
	4 years old		
Passers	*n* = 20 74.1%	*n* = 11 40.7%	*n* = 27 100%
Random	*n* = 1 3.7%	*n* = 3 11.1%	*n* = 0 0%
Non-Passers	*n* = 6 22.2%	*n* = 13 48.1%	*n* = 0 0%
			Total = 27
	5 years old		
Passers	*n* = 22 84.6%	*n* = 17 65.4%	*n* = 26 100%
Random	*n* = 1 3.8%	*n* = 1 3.8%	*n* = 0 0%
Non-Passers	*n* = 3 11.5%	*n* = 8 30.8%	*n* = 0 0%
			Total = 26

The second analysis showed significant age differences ($\chi_{\left(2\right)}^{2}$ = 15.3; *p* < 0.0005) in the proportion of Passers and Non-Passers in the FBT. Detailed analyses showed that the proportion of Passers and Non-Passers significantly differed between 3- and 4-year-olds ($\chi_{\left(1\right)}^{2}$ = 5.12; *p* = 0.02) and 3- and 5-year-olds ($\chi_{\left(1\right)}^{2}$ = 13.04; *p* < 0.0004). There was no significant difference between 4- and 5-year-old children (*p* > 0.05). As expected, the majority of the youngest children were Non-Passers, and the majority of the oldest children were Passers. Significant differences were not found in the proportions of Passers and Non-Passers between the three age groups on either the TB task or the NB task (all *p* > 0.05), and the majority of children were Passers.

Additionally, there were significant differences between age groups on the Test of Language Development results (*F*_(2,67)_ = 25.21; *p* < 0.001). The number of correct answers increased with age. Three-year-olds (*M* = 16.88; SD = 5.21) had more difficulties understanding grammatically complex sentences than did 4-year-olds (*M* = 23.25; SD = 4.61; *p* < 0.001) and 5-year-olds (*M* = 27.03; SD = 4.10; *p* < 0.001). Such a discrepancy was also visible between 4-year-olds and 5-year-olds (*p* = 0.011). Similarly, we observed significant between-group differences (*F*_(2,67)_ = 10.01; *p* < 0.001) in the levels of executive functions. Three-year-olds (*M* = 16.35; SD = 3.28) did significantly worse than older children at response inhibition (*p* = 0.001). However, there was no such effect in the comparison between 4-year-olds (*M* = 18.85; SD = 1.74) and 5-year-olds (*M* = 19.19; SD = 1.54; *p* > 0.05).

Finally, there was a positive correlation between the level of accuracy on the FB condition and the results on both the Test of Language Development (*r_s_* = 0.43, *n* = 70, *p* < 0.05) and the Day–Night task (*r_s_* = 0.28, *n* = 70, *p* < 0.01). Such an effect was not observed for accuracy levels in the TB and NB conditions (*p* > 0.05). When we controlled for the influence of age, only the relationship between the level of language competence and the result on the FB condition remained significant, although slightly weaker (*r* = 0.27, *n* = 67, *p* = 0.024).

### Discussion Study 1

As expected, on the behavioral level, the results of our task reflect the typical pattern of preschoolers’ performance on the classic behavioral FBT. The proportion of Random to Non-Passers and Passers in the FB condition indicates that the observed distribution of responses is not accidental. While most 3-year-olds consistently ignored the character’s FB, almost half of 4-year-olds and majority of 5-year-olds appreciated the character’s FB, avoiding reality bias. The ToM task’s results are therefore highly consistent with previous research demonstrating that the developmental breakthrough in explicit ToM takes place between the ages of 3 and 5 (Wellman and Cross, 2001). The proportion of Passers in FB trials increases with the participants’ age while in the TB and NB conditions was relatively high and stable independently of the age group. These results are, therefore, in line with the all-or-none model: a child is either capable of FB reasoning or not. In the case of the current study, the total number of children classified as Random was significantly smaller than would be expected in the case of random distribution. Only six out of 75 children were classified as responding randomly. Assuming that at least some of these children may represent the transitional period in FB reasoning (Baker et al., 2016), this result points to the high reliability of our test.

Moreover, the positive correlation of FB understanding with levels of sentence comprehension and inhibitory control illustrates the developmental dependence between maturation of explicit ToM, language, and executive functions (Grosse Wiesmann et al., 2017). However, the fact that the partial correlation between ToM and language was weak (<0.3) and, in the case of ToM and inhibitory control, not significant suggests that the language and inhibitory control content in the authors’ custom version of the computerized explicit ToM task was successfully reduced.

At this stage, we managed to address some of the features of a well-designed FBT specified above. We used custom-designed child-friendly stimuli (feature 1). We also used adequate control conditions as similar as possible to the experimental conditions and differing only in terms of belief induction. We included both TB and NB control conditions in order to address both the specificity and generality criteria of the ToM neural correlates (feature 3). In order to minimize executive demands (feature 4), only one character was included in scenario—the toys moved themselves during the change of location phase and the role of the experimenter was limited to the training session, as the consecutive trials and the recorded test questions were presented automatically. The task reduces linguistic demands as the following events in the plot (e.g., leaving the scene by the protagonist and the change of toy’s location) were introduced with the nonverbal animation. Requiring the explicit but nonverbal answer, i.e., pointing at the touch screen, was also aimed at weakening the linguistic demands of the task (feature 5). The all-or-none pattern of results and the progression of performance between younger and older participants suggest that we have managed to create a tool resistant to repeated trials, which is necessary in neuroimaging studies (feature 6).

Studies 2 and 3 aimed to test whether our ToM procedure recruits the ToM network in the expected pattern.

## Study 2

### Materials and Methods

#### Participants

A total of 16 right-handed adults participated in the study. Three participants did not complete the experiment due to technical problems. The final sample consisted of 13 adults (eight females; aged 22–39 years, *M* = 29). All participants had normal or corrected-to-normal vision, did not have any reported history of neurological disorders, and gave written informed consent prior to the study. They were recruited through web-based announcements.

#### Functional MRI Data Acquisition

fMRI data were acquired at the Biomedical Imaging Center of the IPPH in Kajetany/Warsaw, Poland. RS-fMRI examination was conducted using a 3 T Siemens TRIO TIM scanner equipped with a 12-channel head matrix coil. The parameters of the EPI sequence were: time of repetition (TR) = 2,500 ms, time of echo (TE) = 30 ms; flip angle (FA) = 90 degrees; voxel size = 3 mm × 3 mm × 3 mm; imaging matrix = 64 × 64; number of slices = 46; time of acquisition (TA) = 10.4 min; 250 data points. A structural T1 MR sequence had the following parameters: TR = 1,900 ms; TE = 2.3 ms; time of inversion TI = 900 ms; FA = 9 degrees; field of view (FOV) = 256 mm; voxel size = 0.9 mm × 0.9 mm × 0.9 mm; number of slices = 208; TA = 5.09 min.

#### Functional MRI Paradigm

The general fMRI procedure was analogous to the one in the behavioral part of the validation, but the subsequent elements accrued faster than in the behavioral one as we assumed that adults would require less time to process information. The scheme of the procedure was as follows: in each condition, the character/color-matched rectangle leaves the scene in fourth to seventh second; comes back between seventh and ninth second in TB and NB conditions; the toy moves itself from one box to another, and the lids are closed again; in FB condition, the character comes back in the 12th second. Finally, the previously recorded test question is introduced in the 13.5th second. In each condition, the scene is displayed for 5 s and after that period of time, the next trial starts automatically. Each block lasts 18.5 s.

Each run consisted of six blocks of FB, TB, and NB conditions and 15-s fixation periods (a sun) at the beginning of the run and after each block. Each participant performed two runs. The total fMRI experiment lasted 20.1 min.

Participants lay in the MRI scanner while watching short animations and had to press a button indicating their responses to the questions (the questions were the same as in the behavioral part of the study) or passively view a picture of the sun. The animations were displayed using a binocular video display (Nordic Neurolab Inc.™).

#### Functional MRI Data Preprocessing and Analysis

A standard preprocessing pipeline was applied using the Statistical Parametric Mapping 12 (SPM12) software^1^. Preprocessing of the functional data included slice timing correction, motion correction, band-pass filtering (256 Hz), coregistration to individual T1 structural scans, spatial normalization to MNI space, and spatial smoothing (6-mm Gaussian kernel). Each subject’s structural scan was segmented into gray matter, white matter, and cerebrospinal fluid (CSF) tissue classes using the unified segmentation approach implemented in SPM12.

For the subject-wise analysis, individual subject first-level models were created using a general linear model with conditions (FB, TB, NB, fixation period) as covariates of interest. Second-level random effects analysis was performed on the contrast images generated from the first-level models for FB > NB; FB > TB; TB > NB. The whole-brain contrasts were family-wise error correction (FWEc) corrected for multiple comparisons at *p* < 0.05. Data were visualized using the MRIcroGL toolbox^2^. Automated Anatomical Labeling (AAL) Atlas was used to localize brain activations.

All trials were included for the analysis as adults (as expected) found the task simple. The task accuracy for each condition was as follows: NB—91%; TB—84%; FB—79% correct answers.

### Functional MRI Results

**Figure 2:**
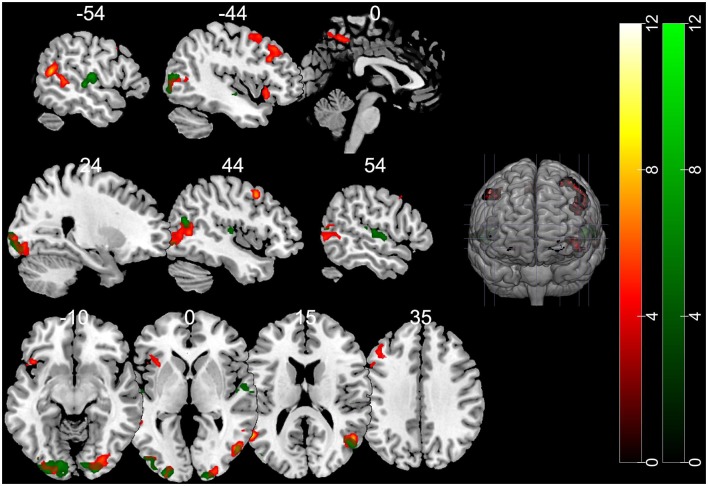
Activation maps for the contrasts false belief (FB) > no belief (NB; hot) and true belief (TB) > NB (green). All clusters were significant at a cluster-forming threshold of *p* < 0.001 and family-wise error correction (FWEc) of *p* < 0.05.

The contrast of the FB relative to the NB condition revealed activity in regions implicated in belief attribution: the posterior parts of medial and superior temporal sulcus bilaterally, precuneus, middle frontal gyrus bilaterally, left inferior frontal gyrus (see Schurz et al., 2014; Schlaffke et al., 2015; Molenberghs et al., 2016), as well as regions associated with visual perception and more specifically face perception: inferior occipital gyrus and right fusiform gyrus (respectively). All regions are presented in Figure 2 and Table 2 (*p* < 0.001 and FWEc of *p* < 0.05; number of voxels >103).

**Table 2:** fMRI BOLD activations for contrasts of interest (whole-brain analysis).

Contrast	Region	Number of voxels	Peak coordinates MNI	Peak *t*
			*x y z*	
FB > NB	Occipital_Inf_R/Fusiform_R	490	30 −84 −14	8.33
	Occipital_Inf_L/Fusiform_L	425	−34 −84 −4	6.44
	Frontal_Inf_Orb_L	103	−42 18 −8	6.06
	Superior Temporal Gyrus/Temporal_Mid_R	448	50 −60 14	8.13
	Superior Temporal Gyrus/Temporal_Mid_L	311	−64 −44 6	11.45
	Frontal_Mid_L	420	−32 10 60	6.33
	Frontal_Mid_R (aal)	132	46 10 48	9.57
	Precuneus_R	134	2 −54 52	6.04
TB > NB	Occipital_Mid_L. Fusiform_L	838	−26 −96 −4	8.30
	Lingual_R (aal)/Cuneus	333	20 −94 −8	7.19
	Temporal_Mid_R/Superior Temporal Gyrus	252	50 −66 18	9.25
	Temporal_Sup_L	215	−54 −22 2	7.71
	Temporal_Sup_R	153	52 −16 8	6.15
FB > TB*	Temporal_Mid_R	11	50 −40 2	4.68
	Temporal_Mid_L	23	−56 −56 22	5.06

*This contrast did not survive correction for multiple comparisons. BOLD, blood oxygen level-dependent; fMRI, functional MRI.

The TB vs. NB contrast showed similar activation in the posterior right STS/MTS to the FB vs. NB contrast, suggesting that both conditions (FB and TB) required mentalizing inference. However, there was no increased activity in the frontal regions or precuneus. All regions are presented in Figure 2 and Table 2.

Nevertheless, direct comparison of neural activation associated with an FB minus TB identified greater activity in the middle temporal gyrus (MTG). This may suggest that the FB condition requires more computations of mental representations. However, this contrast did not survive correction for multiple comparisons.

### Discussion Study 2

A whole-brain random-effects analysis of BOLD response for FB > NB and TB > NB conditions revealed brain activation typically found in explicit FBTs: the middle prefrontal cortex, precuneus, and posterior middle and superior temporal cortex (for a review see Schurz et al., 2013, 2014; Molenberghs et al., 2016). Previous studies have found that the activation of the middle prefrontal cortex reflects the inhibition of one’s own irrelevant perspective when making visual perspective judgments of others (for a review see Schurz et al., 2013). It has been suggested that the precuneus is involved in mental imagery representing the perspective of another person. Finally, the posterior middle and superior temporal cortex is linked to simulating the mental states of others (Beauchamp, 2015).

The additional activation in the occipital cortex and FFA was beyond the scope of our interest. Nonetheless, it probably resulted from the fact that both FB and TB conditions involved the presence of a human-like character, absent in the NB condition. Also, as has been demonstrated in other studies, activation of the visual cortex is modulated by attention (Posner and Gilbert, 1999). In the current work, the increased activation of these areas could possibly reflect the greater demands on attention in the case of belief attribution conditions.

Importantly, we observed overlapping activity in FB and TB conditions in the right posterior MTG/STS. These regions are consistently activated across different ToM tasks and modalities (videos, animations, cartoons; Schurz et al., 2013; Molenberghs et al., 2016) and overlap with the ventral part of the posterior TPJ (pTPJ; Mars et al., 2012). According to previous studies, the TPJ is not a unitary area but encompasses functionally heterogeneous subregions expanding from the lateral occipital cortex through the posterior STS up to the inferior parietal lobule (Mars et al., 2012). Specifically, the posterior MTG/STS, activated here by FBT and TB task, corresponds to a subregion of the pTPJ which is considered a hub for mentalizing and, according to a connectivity-based subdivision of TPJ, shows functional coupling with other regions prominently implicated in social cognition: precuneus, middle frontal gyrus (Mars et al., 2012). These regions also showed increased activity in response to our custom ToM task.

We designed the fNIRS procedure on the basis of the results of the fMRI study described above.

## Study 3

### Materials and Methods

#### Participants

A total of 19 children aged 3–5 years recruited through web-based announcements took part in the fNIRS part of the validation. Written informed consent was obtained from the parents/caregivers of the children participating in the study. All children assented to taking part in the experiment. The final dataset consisted of nine of these children (aged 3.7–5.11 years), as 10 were excluded from further analysis due to a low signal quality caused by excessive motion or insufficient optode adhesion. The latter was partly because the NIRS system used in the study was optimized for testing younger children.

#### Functional Near-Infrared Spectroscopy Measurements

A Gowerlabs NTS Optical Imaging System (continuous wave) system was used to acquire the BOLD signal in children. It consisted of 32 sources (2 × 16, 780- and 850-nm infrared laser diodes) and 16 light detectors (avalanche photodiodes). Measurements were made at a sampling rate of 10 Hz. Optodes were placed bilaterally. The tissue of the temporoparietal cortex, superior temporal sulcus, and dorsolateral prefrontal cortex was covered by the headgear plugins (Figure 4). The custom-made optode array was developed based on the results of an fMRI study using the same stimuli as well as a literature search of common brain areas engaged in FBTs and visual perspective-taking (for a review see Schurz et al., 2013, 2014).

**Figure 3:**
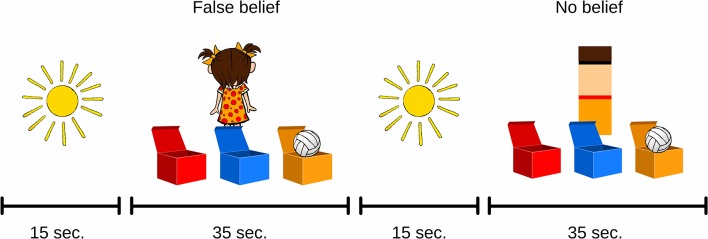
Examples of ToM tasks used in the functional near-infrared spectroscopy (fNIRS) procedure.

**Figure 4:**
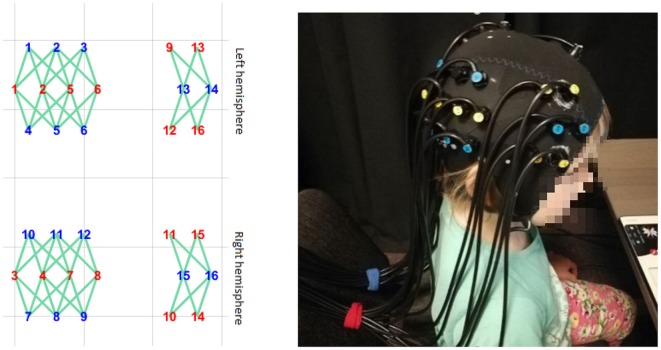
The headgear’s placement on the subject’s head. The layout was designed to cover the temporoparietal cortex, superior temporal sulcus, and dorsolateral prefrontal cortex.

Optodes were arranged into separate rows, with 2.7-cm separations between the rows and 2-cm separations between optodes within each row. Additionally, optodes in two consecutive rows were displaced 1 cm to the left or right to form a rhomboid pattern so that sources in the middle row would reach up to eight detectors in the neighboring rows. This array design allowed us to increase the number of optical channels covering the tissue and to differentiate the depth of the channels. In order to maintain fixed locations within the array, optodes were inserted into the layer of elastic silicone, which was fixed within the elastic cap [based on an EEG cap (EASYCAP™) with no electrode sockets].

#### Functional Near-Infrared Spectroscopy Procedure

The general procedure with the use of fNIRS was similar to the one in the behavioral part of the validation. As a result, the fNIRS ToM task consisted of four FB trials and four NB trials (Figure 3). All trials were separated by a fixation phase of 15 s in which a static illustration of the sun was displayed. This allowed us to register a sufficient amount of BOLD signal change samples within the FB and control NB conditions and to ensure the adequate statistical power of further analyses. The fNIRS part of the testing was done in the NIRSLab at the Faculty of Psychology, University of Warsaw. The headgear was placed on the child’s head before the training session. The training session was identical to the behavioral task. First, initial measurements of signal strength and quality were taken. Any channel with an unsatisfactory attenuation value was adjusted by displacing the disturbing hair and measuring signal parameters once again before starting the procedure. Once the ToM task was administered, the headgear was taken off and the participants continued to perform behavioral tasks, including the Day–Night procedure and the Test of Language Development.

#### Functional Near-Infrared Spectroscopy Data Reduction and Analysis

fNIRS data were analyzed using HOMER2, a set of freely available Matlab scripts (Homer2 v2.8; for a review see Huppert et al., 2009). First, in order to determine which data were invalid because of the motion of the subject, the raw signal was visually examined and portions of data were manually excluded. Raw data were pruned using an algorithm which excluded channels with mean light density which was too low (<0) or too high (>1 × 10^7^) or had insufficient signal-to-noise ratio (<2).

Subsequently, data were converted into ΔOD units, and wavelet analysis was applied. In order to reduce high-frequency instrument noise and biological or motion artifact noise, low-pass (0.25 Hz) and high-pass (0.01 Hz) filters were used.

**Figure 5:**
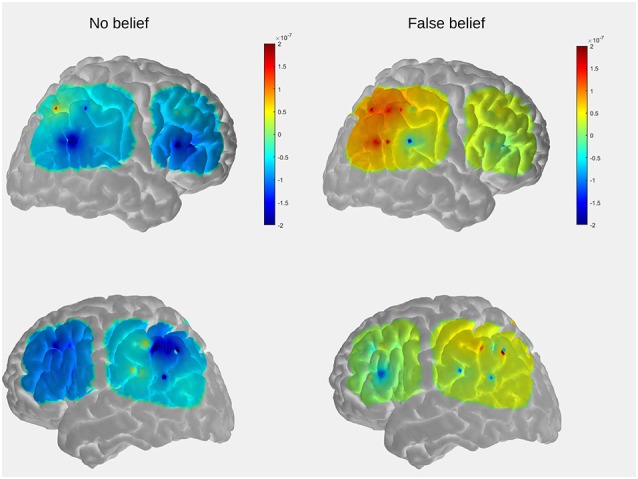
The comparison of mean group results for the location-change period between conditions within the temporal, parietal, and prefrontal regions. The peak activation is observed in the posterior part of the right lateral array.

Statistical fNIRS data analysis focused on the change in concentration of oxygenated hemoglobin (OxyHb), which is considered to be a reliable measure of functional brain activation in fNIRS research (Strangman et al., 2002). However, to provide complete information, deoxygenated hemoglobin (DeoxyHb) time course was also included in visualization of concentration change (Figure 5). Data from all eight trials (four per condition) were included in the analysis.

First, the average OxyHb response was extracted from the specific time period containing the main events: the change of the toy’s location, question, and decision time (13–30 s for the FB condition, 17–33 s for the NB condition). We chose the beginning of the relocation event as a starting point for the time window of interest as previous fNIRS research conducted by Hyde et al. (2018) in preverbal infants demonstrated that OxyHb increases already during the relocation phase, suggesting spontaneous belief tracking. As, we did not attempt to discern the implicit from the explicit ToM process (which will be the aim of our future studies), we decided to include the whole period when mentalizing (spontaneous and deliberate) might occur.

Then, paired t-tests were used to compare the average OxyHb concentration between FB and NB conditions and indicate whether there were any significant differences in OxyHb concentration between the 16 pairs of contiguous channels.

### Behavioral Results

In a group of nine participants who made up the final dataset, all subjects performed well in the NB condition (*M* = 3.9, SD = 0.3). In the FB condition, there was only one participant who scored less than 2 points, other participants were assigned to the group of FBT Passers (six subjects) or Random performance (two subjects; *M* = 3, SD = 1.1).

### Functional Near-Infrared Spectroscopy Results

**Figure 6:**
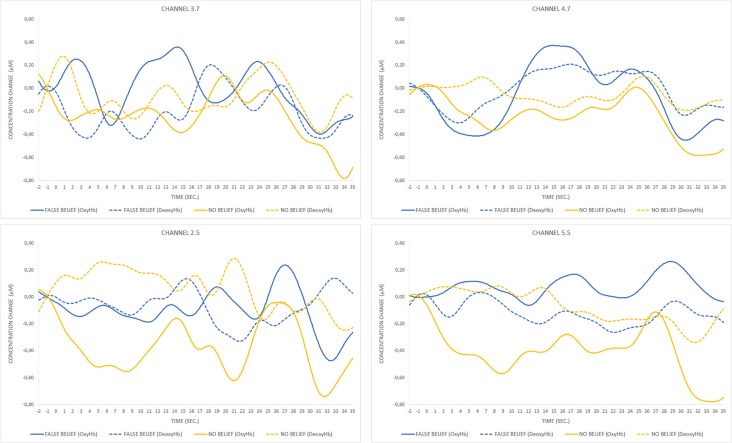
Time course of oxygenated hemoglobin (OxyHb) and deoxygenated hemoglobin (DeoxyHb) concentration change for pairs of significant channels over FB and NB trials. The peak of OxyHb concentration in the FB condition for channels 3.7 and 4.7 located in the right temporal region corresponds to the timing of the toy’s location transfer (13–19 s) and is not observed during the same event occurring in the NB condition (17–23 s) or any condition within the pair of channels located in the left hemisphere.

For the FB condition, a significant increase (*t*_(7)_ = 3.2, *p* = 0.015) in OxyHb concentration (μMol) for the analyzed period was observed in a posterior part of the right lateral array (channels 3.7 and 4.7), which is centered approximately over the right posterior temporal regions. The second significant result (*t*_(6)_ = 3.8, *p* = 0.009) was obtained for the pair of channels (2.5 and 5.5) located in the left lateral array, approximately over the left parietal and superior temporal regions. The time course of OxyHb and DeoxyHb concentration change for channels 3.7, 4.7, 2.5, and 5.5 over the entire trial duration for both FB and NB conditions is presented in Figure 6.

For the purpose of visualization and interpretation of data, the group average OxyHb concentration was displayed on a generic head model using the open source AtlasViewer software (Aasted et al., 2015). In the first step, the probe geometry was positioned on a high resolution Colin27 atlas and registered to the surface. The positions of five key reference points (Nz, Al, A2, Cz, Iz) were obtained with a three-dimensional (3D) scanner from one of the subjects (a 5-year-old boy) to determine the transformation to the digitized space. The preliminary results, obtained to serve as an example, reveal that the temporal and parietal regions which show the strongest activation in the FB condition partially overlaps with those observed in the fMRI study in adults (see Figure 2 in “Functional MRI Results” section).

### Discussion Study 3

The goal of the third study was to check whether it was possible to effectively monitor FBT-specific brain activity in preschool children using the fNIRS technique.

Data analysis focused on differences in levels of OxyHb concentration averaged for pairs of contiguous channels. Higher levels of OxyHb for FB over NB trials were observed in channels located approximately over the right posterior temporal regions.

The comparison of OxyHb concentration time courses between conditions revealed different waveform patterns, with the peak activation occurring during the toy’s relocation phase within the FB condition exclusively. A similar pattern of activation has previously been observed in fNIRS research in adults (Hyde et al., 2015) and 7-month-old infants (Hyde et al., 2018) in which participants free-viewed video clips in a transfer-location paradigm containing FB, TB, and direct perception conditions. In that case, significant differences between conditions arose only during the relocation phase in the FB condition, suggesting that OxyHb concentration in the rTPJ increased after the introduction of conflict between the agent’s belief and the toy’s actual location. The current study revealed that an analogous pattern can be observed in explicit, verbal ToM tasks. As, we managed to collect the behavioral responses of children and the majority of them succeeded in the task, it is highly likely that observed activity over the posterior temporal regions indeed reflects mentalizing. Although the major goal of the current study was methodological, our pilot results seem consistent with few studies of neural correlates of ToM in preschoolers (Gweon and Saxe, 2013; Richardson et al., 2018), suggesting that a key component of the neural organization underlying ToM (rTPJ) is already involved in tracking mental states of others at that age.

The results obtained in the fNIRS experiment are, to some extent, consistent with those found in the fMRI part of the study. In both cases, the higher activation in the FB condition was observed in the posterior superior temporal cortex, which lies within the TPJ, suggesting that common neural structures from the ToM network are engaged in the explicit belief processing elicited by our version of FBT in both children of preschool age and adults. This confirms that fNIRS may be a useful technique for testing ToM in preschoolers.

Together with Studies 1 and 2, Study 3 provides evidence that our custom ToM task is equally well-adjusted to computerized behavioral testing and neuroimaging techniques (feature 2).

## General Discussion

Previous behavioral research has increased our knowledge about ToM development. However, studies which aim to integrate behavioral indicators of ToM development with its neural underpinnings still remain scarce and are usually limited to children older than 6 years.

The main purpose of our study was methodological. We attempted to integrate the behavioral and neuroimaging approaches in ToM research by overcoming limitations present in other studies—the lack of selectivity and lack of controlled tasks—by creating a well-controlled, child-friendly FBT. We minimized executive demands by expanding the training session, limiting the role of the experimenter during the testing, and introducing only one protagonist to be followed. In addition, we precisely adjusted experimental (FB) and control conditions (TB, NB).

The usability of the task for further application in neurodevelopmental research was tested with three methods: first behaviorally with the use of a touch screen on a group of 75 children, then with fMRI measurements on 16 adults, and finally with fNIRS on 19 children.

In line with our expectations regarding the behavioral part of the study, the participants’ performance on the custom version of ToM task corresponded to the all-or-none model. We managed to prove that custom adaptation of the FBT efficiently differentiates the sample, with the FB condition remaining the most demanding one. Most of the participants successfully dealt with our control conditions (TB and NB), as there were no significant differences in accuracy between the three age groups (3-, 4-, and 5-year-old children). Moreover, the accuracy of the children’s answers in the FB condition increased with age and was positively correlated with their level of language development. Therefore, most of the behavioral results support our hypotheses and therefore fulfill the first of our indicators, proving that our task is a reliable tool for computerized behavioral testing of explicit ToM in preschoolers.

On the neural level, we assumed that greater BOLD signal changes will be observed within cortical regions of the ToM network for the belief conditions, with the highest response being for FB trials. The fMRI data analysis revealed greater activation within structures commonly assigned to the ToM network, such as posterior parts of medial and superior temporal sulcus bilaterally, precuneus, middle frontal gyrus bilaterally, and left inferior frontal gyrus. This meets the assumptions of our second indicator.

According to our third assumption, a comparable pattern of neural responses should be observed across the two imaging modalities (fMRI and fNIRS) in the two groups of subjects: preschoolers and adults. The fMRI and fNIRS results support this hypothesis: the custom version of the visual ToM task in both imaging modalities elicited a neural response within the ToM network and therefore constitutes a reliable tool for further ToM research.

### Limitations

Some disadvantages of the custom FBT should be pointed out. As far as the stimuli are concerned, the animations were composed of simple 2D pictures. Protagonists’ movements were therefore rough displacements to the right or to the left. The lack of subtle changes of head position, facial expression, or gaze could have decreased the ecological validity of the tasks and made it more difficult for the participants to engage in mentalization. On the other hand, children usually anthropomorphized the rectangle which replaced the human character in the NB condition, claiming that it hears or sees something. The results of neuroimaging did not, however, reveal any activations, suggesting that the processes of mentalizing occurred during NB events.

Moreover, we obtained almost ceiling results in the Day–Night task. This might be the reason why there was no significant relationship between the level of reaction inhibition and accuracy on the ToM task after controlling for age.

In the fMRI study, we collected data from 13 participants only. However, we observed a pattern of activation pertaining to the ToM network that is similar to other studies directly investigating neural correlates of belief inferring (Schurz et al., 2014; Schlaffke et al., 2015; Molenberghs et al., 2016). Therefore, we concluded that we successfully managed to validate our custom task, and further studies on adults are not needed.

In the fNIRS study, we did not collect exact measures of head topography from each participant. The estimated coverage of layout was therefore imprecise and could have contributed to the variation between subjects.

Almost 50% of participants in the fNIRS study were not included in the analysis due to insufficient signal quality, caused by excessive motion or unsatisfactory optode adhesion. The latter was often observed in participants with dark and thick hair. The solution to this problem could be upgrading the system, in particular, the use of optodes that penetrate better between the hair or more powerful infrared laser sources.

### Summary and Future Prospects

Despite the abovementioned limitations, our results indicate that we have managed to develop a task which can be successfully applied in both precise computerized behavioral and neuroimaging studies of ToM. The use of colorful, friendly animated stimuli makes the procedure attractive and engaging for children and therefore suitable for pediatric research. Due to the presence of three fine-tuned conditions inducing both belief (FB, TB) and NB inferences, the task satisfies both criteria of a model FBT: generality and specificity. The potential influence of the experimenter on the child’s performance is minimized as their active participation is limited to the training phase. The main testing part is fully automated.

In addition, after small adjustments (limiting the child’s role to watching the scene passively, without asking a test question and requiring any response), our explicit procedure can be easily changed into an implicit one and can therefore enable effective exploration of implicit and explicit ToM neural correlates with comparable stimuli.

Maintaining an emphasis on the features which we have successfully applied in the current study, we are currently working on an improved version of the ToM task with the use of 3D animations, including naturalistic character movements (breathing, walking, gaze following the change in the toy’s location). The animations will be temporally fine-tuned between conditions, with the location change occurring at exactly the same time. We are planning to eliminate the anthropomorphized rectangle from the NB condition to avoid potential overmentalizing. In order to maximize the quality of signal and to minimize the number of dropouts, we plan to upgrade our NIRS system with spring-loaded optodes, better penetrating hair and providing better adhesion to the scalp. Such refinements may contribute to improving the accuracy of further studies on the cognitive and neurodevelopmental mechanisms of ToM development. The presented custom ToM task (after the aforementioned improvements) will be further used to examine the neural underpinnings of implicit and explicit ToM in preschoolers.

## Data Availability Statement

The datasets generated for this study are available on request to the corresponding author.

## Ethics Statement

The studies involving human participants were reviewed and approved by The Committee for Ethics of Research at the Faculty of Psychology, Warsaw University. Written informed consent to participate in this study was provided by the participants’ legal guardian/next of kin. Written informed consent was obtained from the minor(s)’ legal guardian/next of kin for the publication of any potentially identifiable images or data included in this article.

## Author Contributions

MH and AP proposed the general design. AP, MH, and JW designed the behavioral studies. MH, AP, JW, and TW designed the NIRS procedure. AP and TW designed the fMRI procedure. JW, KG, AP, MH, and TW analyzed the data. KG, JW, AP, and MH prepared the manuscript. BK programmed the procedures. JW prepared the graphic materials.

## Conflict of Interest

The authors declare that the research was conducted in the absence of any commercial or financial relationships that could be construed as a potential conflict of interest.

## Footnotes

^1^http://www.fil.ion.ucl.ac.uk/spm/

^2^https://www.mccauslandcenter.sc.edu/mricrogl/
